# Mobile enhanced prevention support for people leaving jail: examining smartphone app integration with peer mentors and contingency management for a population at risk of HIV

**DOI:** 10.1186/s12889-025-26096-4

**Published:** 2026-01-07

**Authors:** Gabriel Edwards, Luke Murphy, Liza Buchbinder, Nina Harawa, Chunqing Lin

**Affiliations:** 1 Division of General Internal Medicine & Health Services Research, David Geffen School of Medicine at University of California Los Angeles, Los Angeles, CA USA; 2 UCLA Center for Social Medicine and Humanities, Semel Institute for Neuroscience and Human Behavior, Los Angeles, CA USA; 3 Department of Psychiatry, Charles R. Drew University of Medicine and Science, Los Angeles, CA USA; 4 Department of Psychiatry and Biobehavioral Sciences, David Geffen School of Medicine, University of California, Los Angeles, CA USA

## Abstract

The period following community reentry from incarceration is associated with elevated risks of drug overdose, HIV transmission, and other harms. The importance of leveraging mobile technology to facilitate service access has grown with smartphone use. Peer navigation has shown efficacy for individuals at risk of HIV transmission. We conducted in-depth qualitative interviews with 19 individuals assigned male at birth (all who have sex with men) who were participants in a randomized controlled trial to test an intervention combining the three above components. Three peer mentors were also interviewed. Interviews were focused on the app, GeoPass, and structured using the Health Equity Implementation Framework. Interviewees favorability cited GeoPass’s features that facilitate incentive earning and synthesize suggestions from the peer mentors about local service providers, as well as the ability to track progress to completing personalized goals. Participants expressed willingness to use GeoPass beyond the intervention if it continued to be combined with the peer support and incentive system and suggested additional features to facilitate communication with their peers. GeoPass, as an enhancement of peer support, was generally well-received by the sexual and gender minority participants who had experienced recent incarceration. Peers described the app as helping them manage their clients’ progress towards accessing services and earning incentives. The results highlight the crucial role of peer navigators, when combined with mobile technology, to facilitate smoother societal reintegration and address immediate social needs of people upon reentry from incarceration.

Keywords: mobile health, peer support, contingency management, incarceration, HIV, substance use disorder

**Supplementary Information:**

The online version contains supplementary material available at 10.1186/s12889-025-26096-4.

## Introduction

People experiencing incarceration face multiple health risks including additional risk of HIV and hepatitis C transmission [1]. Formerly incarcerated people are nearly 10 times more likely to be homeless than the general public [2]. The period following incarceration is associated with elevated risk of mortality [3], with drug overdoses among the leading causes of death. Sexual and gender minorities (SGM) experience higher rates of incarceration than the general population [4]. Justice-involved individuals who are also SGM have higher burdens of substance use than non-SGM individuals [5, 6]. Furthermore, those SGM who identify as Black or Latinx face additional structural barriers to accessing vital services, including pre-exposure prophylaxis (PrEP) for HIV [7, 8]. 

Mobile technology’s potential to facilitate service access has grown with smartphone use, now ubiquitous across racial and ethnic socioeconomic groups in the United States [9]. Among the unhoused population, cell phone ownership was estimated at 94%, and smartphone ownership at 58% in 2017 [10], likely higher in 2024. SGM have been found to use the internet more frequently than the general population [11]. A number of papers have been published about developing mobile apps for HIV prevention for SGM in the United States [12–14], with some showing efficacy in increasing PrEP uptake and HIV testing [15]. A feasibility study testing an app for HIV-negative SGM found that participants favored testing locators, testing reminders, and the app’s health information content [16]. These findings and the widespread use of mobile technology among the target population suggest viability of app-based service navigation interventions for SGM. It may also be critical as more public service agencies require clients to use remote technology to access some services.

Peer navigation support has shown efficacy among individuals living with HIV [17] as well as those at risk of HIV transmission [18]. Peer interventions have been associated with recovery-based improvements for individuals with substance use disorders (SUDs) [19] and with engagement in mental health services [20]. Peer support has also been tested as an intervention to aid individuals leaving incarceration and is recognized as a valuable component of trauma-informed care for people with criminal justice involvement [20] and a strategy to reduce social isolation [21]. Although peer support is associated with improvements in HIV care engagement post-release [22], a literature gap remains for interventions that provide HIV-negative SGMs with peer support during reentry.

To our knowledge, few studies have examined interventions to improve service linkage for a population at high risk of HIV that integrates a mobile app with peer support, education, and contingency management, the latter of which has been used for SUD and medication adherence [23]. This paper describes experiences of the Mobile Enhanced Prevention Support (MEPS) Study, a randomized-controlled trial designed to facilitate re-entry processes and engagement in HIV services for SGM with SUD in Los Angeles County. The MEPS intervention recruited participants from November 2019 to December 2022 and utilized three evidence-based interventions; participants were matched with a trained peer mentor whose lived experience mirrored the participants’ own, a smartphone app (named GeoPass) to facilitate finding service providers, earning incentives, and tracking personalized wellness goals, the latter of which was developed at the beginning of their participation. The final component was the incentives themselves. The study team developed GeoPass with extensive community input. To receive incentives, participants were required to record appointments in the app, and after the appointment time/date had passed, required to fill out a survey about their experience which was reviewed and the activity verified by their assigned peer mentor. The intervention included $12 incentives for SUD treatment engagement and $18 for meeting with their peer mentor and accessing services, including (but not limited to) establishing care with a primary care provider, being prescribed and initiating PrEP, preventative screenings for HIV, hepatitis C virus, and sexually transmitted infections (STIs), and accessing social services such as job training, psychotherapy, and enrolling in Medicaid. A participant could earn up to $600 in incentives. The act of making appointments in GeoPass was distinct from the need to scheduling appointments with the providers themselves; for example, calling a clinic to schedule a test. The peers had a separate app interface to review participants’ activity and their progress to meeting personalized wellness goals [24], developed with a member of the study team based on their responses to a baseline quantitative survey. The goals could be medical or non-medical in nature, but participants were guided to frame them as specific, measurable, achievable, relevant and timebound [25], including intermediate steps for each one.

This paper describes the interplay between the MEPS intervention components, using qualitative data collected from study participants and peer mentors. We wanted to learn whether the app reinforced the work between the participant and the peer and how it facilitated earning incentives for accessing services and achieving personalized wellness goals. We also wanted to know how app use was influenced by social/contextual factors, such as housing, employment, income, and internet connectivity.

## Methods

### Study participants

In-depth qualitative interviews were conducted with 22 participants, including 19 who received the intervention (referred to below as “MEPS participants” or “participants”) and 3 peer mentors. All were explained the risks and benefits of participation and provided informed consent to participate. Interviews occurred between March and September 2023. Study investigators contacted current and prior MEPS participants and peer mentors to gauge interest and willingness/ability to provide informed consent to participating in semi-structured interviews. To ensure comprehensiveness of data, we enrolled MEPS participants from varied social-demographic backgrounds, incarceration histories, and health statuses.

### Data collection

Interviews were conducted virtually via phone or videoconference. The median interview length was 43.5 min (range 24–65 min). The semi-structured format followed the Health Equity Implementation Framework, designed for implementation researchers to study innovations in the context of health equity determinants and user perspectives [26]. The main domains of the Health Equity Implementation Framework are recipient factors, innovation factors, provider factors, and social/contextual factors. Study investigators conducting interviews included an MD/MPH who studies HIV prevention among criminal justice-involved individuals, an MD with a PhD in medical anthropology who treats patients in the local jail system, and a UCLA medical student with experience conducting qualitative research. Investigators used separate interview guides for participants and peer mentors, both of which addressed the same framework domains. Interview guides developed for this study haven’t been published elsewhere. (Supplementary File 1)

Recipient factors centered on MEPS participants and their health and social needs, including the use of the internet and devices, self-efficacy with technology, the type of apps used regularly, and motivations for behavior change. Innovation factors related to the app itself. Interviewers asked about the appropriateness/ease of use of GeoPass, the reasons for non-use, the users’ favorite/least favorite app features, and how GeoPass app compares to other apps they used. Provider factors focused on the relationship between participants and peer mentors, including desired peer mentor characteristics and how GeoPass facilitated or impeded the peers’ ability to help participants access services and achieve goals, including their ability to orient intervention participants to the app. Interviews also covered social/contextual factors, including housing instability, transportation, access to smart phone and data plans, and social support.

### Data analysis

The recordings were transcribed verbatim and transcripts were uploaded into ATLAS.ti for coding and analysis. Codes were developed based on the Health Equity Implementation Framework and the initial interview guide, with additional codes generated from emergent findings. The investigators collaboratively coded the first few transcripts to ensure intercoder reliability and reconciled differences in coding via discussion. After coding, we reviewed excerpts from each code to conceptualize the nature of the interviewee’s responses.

## Results

### Participant characteristics

#### MEPS participants

Table 1 lists characteristics of MEPS participants who sat for in-depth interviews. The majority reported living in facilities that offered services such as “residential treatment”, “residential bridge housing”, “sober living”, and “transitional housing”. One person reported being unhoused and living with friends, and another reported living in a shelter for the unhoused. Two participants reported living in rented apartments. Eight participants reported being employed at the time of the interview, with two of those being employed as a manager at the facility in which they also lived. Some reported that their facility restricted their ability to go out. Seven participants reported income insufficient for their basic needs. No participants reported unexpected encounters with law enforcement subsequent to enrollment in MEPS, and most participants on parole or probation reported that the terms did not pose restrictions for them. One participant did report losing income because of their parole arrangement. *“I would do some adult work*,* both legal and not*,* but then parole caught on at least to the legal part and banned me from doing it.”* (trans woman, Hispanic, 31 years old). Several participants reported internet connectivity issues with the free smartphone they obtained [27]. However, they still felt that internet access was adequate for their needs.3dxsqa

**Table 1:** MEPS participant characteristics (excl. Peer Mentors)

Characteristic	Total *n* = 19
Age, years, mean, st. dev.	32 ± 7.2
Gender (cisgender male, transgender female), *n*	17, 2
Identified Ethnicity (Hispanic, Non-Hispanic), *n*	9, 10
Identified Race, *n*	
Black	2
Multi-Racial	2
Native American	1
White	14
Housing Status, *n*	
Has own housing	2
Service facility	15
Shelter/unhoused	2
Employment Status *n* (%)	
Employed	8
Unemployed	11
Health Insurance *n*	
Medicaid (Medi-Cal)	12
Private insurance	2
No insurance	5
Legal Status, *n*	
Probation	6
Parole	2
Mental health diversion	1
Both	1
Neither	9

### Features of GeoPass

#### Financial incentives

When asked to discuss their favorite part of the app, participants commonly mentioned the app’s facilitation of incentives. With financial challenges post-release, some participants said that the money earned from MEPS was an important income supplement. *“Like without MEPS*,* I couldn’t afford to pay for shoes. I’m going from just not even making it to be able just to make it.”* (cis male, white, 47). Participants spoke positively of transparency around the incentives. *“I’m able to see a summary of what’s been paid out and what surveys I have remaining. I like that a lot. That really puts things into perspective in terms of what I could still get paid for.”* (Cis male, Hispanic, 32) Some participants reported that their use of the app decreased once they exhausted all their eligible incentives. Peer mentors also liked the app’s incentive tracking function because it allowed them to see what their clients had earned and what possible incentives remained.

#### Progress tracking

In addition to receiving cash incentives, participants also cited the recognition they received as a positive. *“(GeoPass) incentivizes you*,* but after you do something*,* and then you get to go on the app and show that you did it*,* it’s like somebody else knows what you did—that you did it.”* (cis male, Black, 36) Some participants saw incentives as secondary to being able to document their activities and accomplishments, and that their motivation for accessing services evolved to be progressively less incentive-focused. *“I felt like MEPS pushed me into that direction of being more mindful*,* because yeah*,* not only was it incentives*,* but it was something that informed me about how I should be when it comes down to taking care of myself*,* and especially if I’m being sexually active.”* (Cis male, Native American, 35) These two responses support a theme; that the incentives were central to the usefulness of the app, but the ability to track healthy activities was core to maintaining their motivation to improve their health.

#### Goal setting

Participants felt that seeing which preventive or social service activities were incentivized was helpful in thoughtfully communicating the value of the activity. *“The whole idea of GeoPass was ‘How are you bettering yourself? What are you doing to make sure you’re safe? What are your goals?’ That was very helpful.”* (nonbinary/trans woman, multiracial, 26) They also cited the ability to review goals as useful. Like the benefit of seeing their earnings reflected in GeoPass, seeing goals written down helped concretize them. *“Whenever you put something down on paper*,* it makes it more real and more like methodical in your thinking rather than like*,* ‘Oh*,* I just wanna do this*,* and I gotta do this*,* that*,* and the other.”* (cis male, white, 37) The feedback on this feature added to the theme of GeoPass supporting goal achievement for users.

#### Making appointments in GeoPass

GeoPass enabled participants to schedule appointments either before or after receiving a service. “*Well*,* it’s like a notebook of like a—where you could keep your appointments. You could make your appointments or reminders and where to go*,* and then you could go back and be like*,* ‘Okay*,* this is where I went at this time and this place*.” (Cis male, Hispanic, 32) Many participants reported making appointments after completing the service. Appointments made retroactively might have reflected uncertainty about what activities might lead to an incentive until advised so by their peer. *“A lot of it was because I wouldn’t have considered that some of the things I had done were incentivized.’”* (nonbinary/trans woman, multiracial, 26). Not knowing whether an activity would be incentivized reflected a commonly shared view that the in-app appointment process was seen primarily as a means to earn incentives rather than to help them plan the appointments in advance as originally intended. Another participant spoke of how going to appointments would trigger him to think of the app instead of the other way around. *“I knew when I would be at an appointment*,* I would know. I would think*,* ‘GeoPass.’ Your brain automatically thinks that.”* (cis male, black, 36) This was consistent with another finding – that suggestion for the service providers used would often come from the peer mentor, not the app, despite its extensive provider database.

### Peer mentor role

#### GeoPass facilitates peer service navigation

The peers used GeoPass primarily as a tool to help participants track and plan their activities based on incentives. *“When I needed to just go over the percentages or pay*,* the pay scale or the amount of incentive they have received previously or to date*,* it was definitely an asset because you were able to see it in real time exactly what has been incentivized*,* what is still available to be incentivized. It was very beneficial to me.”* (cis male, black, 68, peer mentor).

When asked about how they selected service providers, many participants said that their selection was a suggestion from their peer mentor. As one participant stated, *“[The peer mentor] would look up stuff and send it to me*,* like on the phone. Like*,* ‘Hey*,* call this place. This is where you should go. This is closest to you.’ He would know where my address was at*,* and then he would tell me what’s around there.”* (cis male, black, 36) A peer mentor explained, *“Most of them kind of rely on working directly with us to choose a place for them. They are accustomed to having their hands held through that process of finding a resource.* (cis male, black, 40) They also thought that GeoPass was better for time-limited services such as testing. “*I think that … the services part… is best suited for maybe STI*,* STD*,* and HIV testing. For a lot of my participants who may be looking for work*,* it may not be the best source to find services that will connect them to work. That kind of requires a little more detailed information that the GeoPass app probably doesn’t really provide for them.”* (cis male, black, 40). It was relatively uncommon for a participant to identify providers using GeoPass’s database without consulting their peer mentor, a dynamic of which the peer mentors were very aware.

One peer mentor discussed his own discomfort with technology. *“Well*,* because I wasn’t the tech-savvy guy and computer literate*,* I was actually encouraged by my clients because they were*,* and they could navigate [GeoPass].”* (cis male, black, 68) However, neither peers nor participants reported tech literacy as a major barrier to the use of GeoPass, and this peer mentor reported that he received support from his participants when using the app.

### Other suggestions and feedback

#### Privacy

Interviewers asked participants about their privacy concerns with GeoPass. One participant cited app content containing their HIV testing and attendance of twelve-step programs, but he was an exception. Others were not concerned with privacy risks while using the app, although multiple people added that their privacy was already at risk and GeoPass did not meaningfully add to that hazard. *“I think at this point*,* in all honesty*,* it’s everything’s about as safe as it is. With hackers and everything as it is*,* you have your banking—people typically have their banking information*,* all kinds of information on their phone*,* so why not have your healthcare information as well.* (trans woman, Hispanic, 31) There may have been privacy concerns with the GeoPass app, but they were not generally perceived as greater than the privacy risks of online technology in general.

#### Suggested features not in GeoPass

When asked about additional GeoPass features that they would like to see included, a number of participants stated that they would like the app to better facilitate communication with peer mentors. They suggested adding a function to enable direct messaging with them. In the absence of this feature, participants communicated with their peers using messaging apps outside of GeoPass; peer mentors did not cite difficulty communicating with their participants in this way. Participants suggested including video chat functions as well; peer mentors and participants reported using a blend of in-person, phone, and platforms such as Zoom for their scheduled check-in sessions. This was not a feature in the version they used when participating in the MEPS intervention.

#### Sustainability

Participants expressed hesitation to use GeoPass without the other intervention components in place. *“If this was something that had an ongoing lifespan*,* maybe using it to communicate or to have goals set with my peer would be very helpful. (Interviewer) Can you imagine using it without a peer mentor in any way? Interviewee: Maybe not.”* (nonbinary/trans woman, multiracial, 26) On the other hand, multiple participants stated a desire to continue working with their peer mentor even after they could no longer earn incentives. *“I don’t think that it should matter whether or not I’m getting incentives anymore. I think it’s just having someone that is supportive of my recovery and where I’m going and my experience.”* (nonbinary/trans woman, multiracial, 26) This would suggest that of the intervention components, the peer support had the most inherent sustaining power.

## Discussion

We found multiple layers of interaction between the GeoPass app’s features, the users’ needs, and other aspects of the Mobile-Enhanced Prevention Support intervention. Participants provided significant insights into the factors that affect linkage to care in this population. The SGM participants in this intervention experienced unmet social needs post-incarceration, such as unstable housing conditions, unemployment, and under- or uninsurance. Employment, housing, food security, and health insurance are crucial prerequisites for accessing SUD treatment and maintaining overall well-being following release from incarceration [28, 29]. A drug arrest or charge, even without a conviction, can be a barrier to employment [30]. Considering these social and economic challenges, it is understandable that participants saw monetary incentives facilitated by GeoPass as not just a motivation for engaging in the intervention, but a vital component of their ability to meet their basic needs. The app’s role in facilitating incentives, both in the ability to earn them as well as in the ability to track what could still be earned, came up more frequently than any other feature. Policies to effectively support the community reintegration of formerly incarcerated SGM must encompass employment support and anti-discrimination measures designed to overcome the barriers to employment posed by criminal records. Addressing employment and financial challenges is essential for reducing the vulnerabilities faced by SGM post-incarceration and for promoting equitable service use and health outcomes.

An important theme was the in-app GeoPass features that participants valued beyond incentives, such as documenting goals and monitoring progress towards achieving them. These features are particularly beneficial for managing health and tracking behavioral changes post-incarceration. A study that combined peer navigation with a mobile application found these features similarly valued among participants reintegrating into the community [31]. The ability to track progress in real-time reinforces participants’ commitment to their pre-set milestones and promotes a sense of accomplishment. This encourages regular engagement with the app as a part of their effort to accomplish goals, indicating potential for its sustainability as part of a peer intervention. The input from participants and peer mentors demonstrates that SGM individuals can use GeoPass to aid health improvement and social connectivity, demonstrating a digital health intervention that can comprehensively address both external and internal motivators.

Looking ahead, future enhancements to GeoPass could incorporate more robust features supporting peer-participant communication such as direct messaging, or built-in automated messaging that offers positive reinforcement when participants achieve objectives. Additionally, organizations that provide peer support should also explore ways to transition the peer role to a member of the clients’ own social network to ensure sustainability. This would complement the existing features that track progress in linking users to HIV/STI/SUD services and further address the social vulnerabilities highlighted earlier. Rather than being a standalone app, GeoPass could be a tool used by organizations whose programming includes providing peer support to clients.

Another important theme was the way GeoPass functioned in the context of work with a peer mentor and an incentive system. Participants reported that their interest in using the app was contingent upon the other intervention components, such as the presence of peers and incentives without which the value of the app would be much lower. Peer-assisted approaches are essential for addressing the unmet needs of vulnerable and marginalized populations, including those with mental health and substance use [32, 33]. Peers are themselves on a recovery journey, and so can earn trust and serve as role models for individuals with SUDs, mental illness, or other challenges [31]. The peer navigation approach has received increasing recognition for helping individuals reintegrate into the community and improve their health outcomes post-incarceration [34]. In our study, intervention participants described GeoPass as enhancing peer mentors’ ability to assist them with goal setting, tracking progress, and accessing necessary services. Interestingly, while peer mentors were tasked with orienting participants to the app, in some instances it was the participants who aided their peer mentor in app utilization. This finding highlights the significance of human interaction and mutual support for SGM post-incarceration, with mobile apps like GeoPass serving as a technological tool augmenting these human connections and linkages to care.

This study provides valuable insights into using combined peer navigator-mobile app strategies to engage individuals in health management post-incarceration by describing how a peer mentor, and app, and an incentive structure for engaging in services fit together in the context of the MEPS intervention. Due to high smartphone adoption, mobile app interventions for supporting SGM post-release are accessible, including among those experiencing unstable housing. However, the limits to data plans and hardware reliability for those using government-subsidized smartphones meant some participants had to rely upon available public Wi-Fi services. This population would benefit, then, from efforts to improve mobile data access. Improving subsidized data plans is crucial for low-income individuals, including those recently incarcerated when attempting to access health information, services, employment opportunities, and social activities. That latter is increasingly vital in this post-COVID digital era that has seen increased social isolation.

Considering the essential role of peer support, the integration of instant communication features within these apps could facilitate seamless, direct interactions between users and their peer supporters. Such real-time communication capabilities are likely to enhance the effectiveness of the support provided, fostering more immediate and responsive peer support that can address the needs of SGM as they navigate post-incarceration life.

Several limitations of this study warrant attention. This study was conducted in Los Angeles, a metropolitan city with an extensive safety net of health resources and robust internet connectivity. The findings may not be generalizable to other regions, particularly rural areas where resources are scarcer and stigma towards the SGM population may differ. Additionally, the potential for social-desirability bias and recall bias could have influenced the participants’ responses to the interview questions given that some were speaking to a member of the MEPS intervention team. This study demonstrated the acceptability of MEPS intervention and highlighted the complex interplay between individuals’ social challenges, technology, and peer support in the post-incarceration experiences of SGM. The results emphasize the urgent need to address the immediate health and social needs of SGMs post-incarceration and the crucial role of peer navigators, when combined with mobile technology, to facilitate smoother societal reintegration.

## Conclusion

GeoPass was generally well-received by SGM experiencing incarceration and their peer mentors who viewed it as a valuable facilitator of other aspects of the MEPS intervention. Peers described the app as helping them manage their clients’ progress towards accessing services and earning incentives. Technology evolves rapidly and can facilitate or complicate other activities. Conducting one-on-one interviews or focus groups with users to understand how mobile technologies are used in their day-to-day lives can help to ensure that their interfaces remain intuitive for the intended user regardless of the included features.

## Supplementary Information

Supplementary Material 1.

## Data Availability

All data from the results of this study are available upon request from the corresponding author.

## Abbreviations

MEPS: Mobile Enhanced Prevention Support
PrEP: Pre-exposure prophylaxis (HIV)
STI: Sexually transmitted infection
SGM: Sexual and gender minorities
SUD: Substance use disorder
