# Correction: Mobile enhanced prevention support for people leaving jail: examining smartphone app integration with peer mentors and contingency management for a population at risk of HIV

**DOI:** 10.1186/s12889-026-26954-9

**Published:** 2026-04-13

**Authors:** Gabriel Edwards, Luke Murphy, Liza Buchbinder, Nina Harawa, Chunqing Lin

**Affiliations:** 1 Division of General Internal Medicine & Health Services Research, David Geffen School of Medicine, University of California Los Angeles, Los Angeles, CA USA; 2 UCLA Center for Social Medicine and Humanities, Semel Institute for Neuroscience and Human Behavior, Los Angeles, CA USA; 3 Department of Psychiatry, Charles R. Drew University of Medicine and Science, Los Angeles, CA USA; 4 Department of Psychiatry and Biobehavioral Sciences, David Geffen School of Medicine, University of California, Los Angeles, CA USA

**Correction: BMC Public Health 26**,** 469 (2026)**

**https://doi.org/10.1186/s12889-025-26096-4**

Following publication of the original article [1], the authors reported an error in Ethics approval and consent to participate section.

The updated Ethics approval and consent to participate is given below and the changes have been highlighted in **bold typeface**.

**Ethics approval and consent to participate**

The study was approved by the Institutional Review Board at University of California, Los Angeles (UCLA) (**IRB#22–0948**) in accordance with the ethical principles of the Belmont Report and federal criteria for IRB approval of research and informed consent. In addition to collecting informed consent from all human subjects, the study adhered to the principles of the Declaration of Helsinki. A clinical trial number is not applicable for this study. Interviewees were provided with detailed information about the study objectives, procedures, risks, and benefits, and gave oral consent to participate prior to data collection.

The original article [1] has been updated.
