# Supplementary material for: Mobile enhanced prevention support for people leaving jail: examining smartphone app integration with peer mentors and contingency management for a population at risk of HIV
Source: BMC Public Health. 2026 Jan 7;26:469. doi: 10.1186/s12889-025-26096-4 (PMC12870299; doi:10.1186/s12889-025-26096-4)
Supplement: Supplementary file 1 — Supplementary Material 1. [file 12889_2025_26096_MOESM1_ESM.docx]

Semi-Structured Interview Guide: Peer Mentor

Topic 1: Health Needs/Risks (Recipient Factors, Societal/Contextual Factors)

***First we’d like to ask you about the most frequent health needs for your clients.***

PROBES

1. How concerned are you clients about housing?
2. Where do you live, and with whom?
   1. Do you have privacy when doing things related to your health (ie, talking to clinics about an appointment, getting advice over the phone)
3. Finding and keeping a job?
4. Having enough money to meet basic needs?
5. Traveling from place to place?
6. Having insurance coverage? What kind of insurance?
7. Have run-ins with law enforcement?
8. Finding health services for substance-related problems?
9. Others?
10. Would you please elaborate if you have challenges with any of these aspects?

Topic 2: Social Needs/Factors (Recipient Factors)

***Now we’d like to ask you about the most frequent social needs for your clients.***

PROBES

1. **How do those needs affect how easy it is to communicate and keep in touch with those clients?**
2. How often had your clients had challenges with staying housed?
3. How did clients returning to jail and then being released affect your work with them?
4. Did your clients experience violence? Run-ins with law enforcement?
5. How did the terms of your clients’ parole/probation affect your ability to work with them?
6. Did your clients have support from anyone in their life besides you? This could include friends, family, people they live and/or work with.

Topic 3: Technology (Recipient Factors)

***Now we’d like to ask you about the role of technology***

PROBES

1. **Did your clients have access to charging for their phones?**
2. **Did they have access to internet?**
3. **How comfortable are your clients with using technology?**
4. If they are uncomfortable, what are the reasons for that?
5. **In your opinion what are some of the strategies to support your client if they have difficulties with technology?**

Topic 4: GeoPass App (Innovation Factors)

***Let’s talk about the app itself, GeoPass. What do you think of GeoPass in general?***

PROBES

1. **How often do you use it?**
2. **What do you use it for the most?**
3. **What are your favorite parts of GeoPass, and why?**
4. **What are your least favorite parts of GeoPass, and why?**
5. **What were *your clients’* favorite parts of GeoPass, and why?**
6. **What were *your clients’* least favorite parts of Geopass, and why?**
7. **Are there any parts you don’t use at all?**
8. **Are there any features you’d like to see that aren’t there (for example: types of services listed, types of health information, community resources)?**
9. Do you like the way it looks (for example, colors, pictures, writing?
10. **Are the parts of the app arranged in a way that does not make sense?**
11. Does it make sense to read?
12. **Do you have any concerns about the app** (probe: privacy)?

Topic 5: Working with Clients (Provider Factors)

***Next I’d like to ask you about your work with your clients. Has GeoPass made it easier or harder to help clients reach services and goals, and why?***

PROBES

1. **Does the app fit in with your working style or not?**
2. How was the experience of orienting participants to GeoPass? After the orientation is over, how much have you had to assist them with the app?
3. **What kinds of problems have they faced with the app? How often had you needed assistance to resolve them?**
4. How much of your work with clients was in person versus remote?
5. When it was remote, what did you use to communicate with them?
6. What were the reasons that determined whether you had in-person or remote meetings?
7. What was the role of GeoPass in clients finding services?
   - How often did they find services through app without you telling them about the services first?

Topic 6: Other feedback (Innovation Factors)

***Finally I’d like to ask if you have any other thoughts about the app. Do you have any other suggestions to improve the app?***

PROBES

1. Do you have any other suggestions to improve access to care for your clients?

***Would you consider using the app in the future role working with clients? Would you be willing to introduce this app to someone you know in a similar circumstances to your clients?***

------------------------------------------------------------------------------------------------------------------------

Semi-Structured Interview Guide: MEPS Participants

Topic 1: Demographics (Recipient Factors)

***First I’ll ask you some basic questions about yourself. You were asked these questions when you enrolled in MEPS but it’s important that we collect them again now.***

1. **How old are you?**
2. **What sex were you given at birth, on your original birth certificate?**
3. **What is your current gender identity?**
4. **What is your race/ethnicity?**
   1. If they need options: American Indian/Alaska Native, Asian, Black/African American, Hispanic/Latino, Native Hawaiian or other Pacific Islander, White, Other, Don’t know, Refuse to answer
5. **Are you on parole or probation right now?**

Topic 2: Social Factors (Societal/Contextual Factors)

***I want to ask you about a variety of things we call social factors. In general, do you have access to the things you need in life on a daily basis?***

- ***Probe: Do any of these things affect your use of GeoPass, or your smartphone, or getting services?***

PROBES

1. Housing?
2. Where do you live, and with whom?
   1. Do you have privacy when doing things related to your health (ie, talking to clinics about an appointment, getting advice over the phone)
3. Finding and keeping a job?
4. Having enough money to meet basic needs?
5. Traveling from place to place?
6. Having insurance coverage? What kind of insurance?
7. Have run-ins with law enforcement?
8. Finding health services for substance-related problems?
9. Others?
10. Would you please elaborate if you have challenges with any of these aspects?

***Now I’d like to ask you about support from social networks. Would you say you have enough of it?***

PROBES

1. This could be from anyone in your life, including friends, family, people you live and work with, etc.

Topic 3: Goals (Provider Factors)

***Now I’d like to ask you about your passport goals. What has it been like working with your peer mentor on your passport goals?***

PROBES

- What are your goals?

Topic 4: Smartphones (Recipient Factors)

***Now I’d like to ask you some questions about your smartphone. Was it challenging to get a smartphone?***

PROBES

1. **Do you have access to charging when you need it?**
2. **Do you have any difficulties accessing internet?**
3. What kind of phone do you use? Is it an Obamaphone?
4. Have you replaced your smartphone since starting with the MEPS study?

Topic 5: Internet Usage (Recipient Factors)

***Next, let’s talk about internet usage. What do you use to access the internet?***

PROBES

1. Examples include smartphone, computer, public wifi, public computers
2. **Are you able to access the internet when you need to?**
3. How much time do you spend on the internet on devices other than your smartphone, like computers?
4. **What challenges do you have with finding information on the internet, if any?**

Topic 6: Smartphone Use (Recipient Factors)

***We talked a little bit about your smartphone earlier. Now I’d like to learn more about how you use it. What types of smartphone apps do you use frequently?***

PROBES

1. **Besides GeoPass, what apps do you use to search for information about health-related questions you have?**
2. How much time do you spend on your smartphone using the internet?

Topic 7: The GeoPass App (Innovation Factors)

***Let’s talk about the app itself. What do you think of GeoPass in general?***

PROBES

1. **How often do you use it?**
2. **What are your favorite parts of GeoPass, and why?**
3. **What are your least favorite parts of GeoPass, and why?**
4. **What are the situations where you are using it the most?**
5. **Are there any parts you don’t use at all?**
6. **Are there any features you’d like to see that aren’t there (for example: types of services listed, types of health information, community resources)?**
7. **What do you think about the way it looks (for example, colors, pictures, writing?)**
8. **Does it make sense when you read it?**
9. Are the parts of the app arranged in a way that makes sense?
10. **Do you have any concerns about the app** (probe: privacy)?
11. Do you have any other suggestions to improve the app?

Topic 8: Peer Mentors (Provider Factors)

***I’d like to ask you about your work with your peer mentor. How has your work with your peer mentor been?***

PROBES

1. **What has been the most helpful parts of working with them? The least helpful?**
2. **How often did you meet with your peer mentor?**
3. **How often did you meet remotely and how much do you meet in person?**
4. When you meet remotely, is it over the phone or using something like zoom?
5. How willing are you to continue working with a peer navigator beyond the scope of the study

Topic 9: Peers and GeoPass (Provider, Innovation Factors)

***Now I’d like to ask you about how your work with the peer mentor and the app fit together. Has GeoPass been a help in working with your peer, getting services, and working on goals, or has it been a barrier? Or neither?***

PROBES

1. Was your peer mentor able to orient you to the app and help you when you had challenges, or have you had to do those things yourself?
2. **How do you think your peer mentor can better assist you to navigate and take advantage of the app?**

***A couple more questions and then we are done:***

1. **In addition to what we have discussed above, do you have other suggestions regarding getting support in getting to healthcare/social services and reach your goals?**
2. **Would you consider using the app in the future? Would you be willing to introduce this app to your friend in a similar circumstance?**
